# Use of botulinum toxin type a in psychiatry - new perspectives and future potential

**DOI:** 10.1192/j.eurpsy.2021.1283

**Published:** 2021-08-13

**Authors:** F. Gonçalves Viegas, I. Figueiredo, F. Ferreira, A. Lourenço

**Affiliations:** 1 Departamento De Saúde Mental, Hospital Prof Dr Fernando Fonseca, Amadora, Portugal; 2 Mental Health Department, Hospital Professor Doutor Fernando Fonseca, Lisboa (Amadora), Portugal; 3 Psychiatry, Hospital Professor Doutor Fernando Fonseca, Lisboa (Amadora), Portugal

**Keywords:** emerging psychiatric indications, Depression, botox, botulinum toxin

## Abstract

**Introduction:**

For almost three decades, botulinum toxin type A (BT-A) has been used for medical purposes. Evidence of the potential use of BT-A is emerging for psychiatric disorders, like unipolar and bipolar depression, borderline personality disorder (BPD), late dyskinesia, amongst others. This may represent a new role of BT-A treatment and could expand the therapeutic arsenal in psychiatry.

**Objectives:**

The goal is to review current evidence regarding BT-A and psychiatry disorders.

**Methods:**

Literature review of BT-A use in psychiatric conditions using Medline database.

**Results:**

There’s evidence supporting the use of BT-A in resistant unipolar depression, with studies showing an 8 and 4 times higher response and remission rates comparing with placebo. Beneficial effects were also found in bipolar depression. Preliminary data suggest that BT-A therapy may also be effective in the treatment of mental disorders characterized by an excess of negative emotions, such as BPD. The underlying mechanism might be the “facial feedback hypothesis”. Hyperhidrosis is a common comorbidity in social anxiety disorder and may itself give rise to depressive or anxiety symptoms. BT-A has proved to be a safe and effective treatment for hyperhidrosis. BT-A can also be safely used for dystonia secondary to the use of psychiatric medication, when there’s an inadequate response to anticholinergic medication. Also, BT-A injections in the salivary glands have been investigated for treating clozapine-induced sialorrhea and studies reported successful reduction in hypersalivation.

**Conclusions:**

Although more studies are needed to evaluate the potential of BT-A in psychiatry, there is growing evidence of its potential use for some psychiatric conditions.

